# Assessment of knowledge and attitude towards palliative care and associated factors among nurses working in selected Tigray hospitals, northern Ethiopia: a cross-sectional study

**DOI:** 10.11604/pamj.2020.35.121.17820

**Published:** 2020-04-15

**Authors:** Teklay Zeru, Hagos Berihu, Hadgu Gerensea, Girmay Teklay, Tewolde Teklu, Haftom Gebrehiwot, Tewolde wubayu

**Affiliations:** 1 Department of Pediatric Nursing, School of Nursing, College of Health Science and Comprehensive Specialized Hospital, Aksum University, Tigray, Ethiopia; 2 Department of Maternity and Reproductive Health , School of Nursing, College of Health Science and Comprehensive Specialized Hospital, Aksum University, Tigray, Ethiopia; 3 Department of pharmacy, School of pharmacy, College of Health Science and Comprehensive Specialized Hospital, Aksum University, Tigray, Ethiopia; 4 Department of Pediatric Nursing , School of Nursing, College of Health Science and Comprehensive Specialized Hospital, Mekelle University, Tigray, Ethiopia; 5 Department of Pediatric, School of Medicine, College of Health Science and Comprehensive Specialized Hospital, Aksum University, Tigray, Ethiopia

**Keywords:** Tigray, attitude, knowledge, nurses, palliative care

## Abstract

**Introduction:**

Palliative care is a multidisciplinary approach which is focused on both the patient and their family. Therefore the objectives of the study is to assess the knowledge and attitude towards palliative care and its associated factors among nurses in Tigray, Northern Ethiopia, 2018.

**Methods:**

An institutional based cross-sectional quantitative study design was carried out using 355 nurses working in selected hospitals in Tigray region from February to March, 2018. Systematic random sampling was used to select six governmental hospitals. We used triangulation in the study method, making use of both Frommelt's Attitude Toward Care of the Dying (FATCOD) scale, and Palliative Care Quiz for Nursing (PCQN) knowledge. SPSS were applied for data entry and analysis. Statistical significance was declared at P<0.05. The goodness of fit the final logistic model was tested by using the Hosmer and Lemeshow test at a value of > 0.05.

**Results:**

All the participants were able to respond. Out of the total study participants, 223 (62.8%) had good knowledge and 200 (56.3%) had a favorable attitude towards Palliative care. A medical ward had (AOR = 3.413, CI = 1.388-8.392, P = 0.019), trained Nurses [AOR = 3.488; CI = 1.735-7.015; P = 0.00) significant associated with nurses knowledge towards palliative care. Nurses working in the lemlem Karl (AOR=2.541; 95% CI; 0.013(1.106-5.835), nurses who had a 20-30 years ago had unfavorable attitude (AOR = 2.660; 95% CI; 0.002(1.386-5.106) were significant.

**Conclusion:**

The nurses had poor knowledge. However, their attitude towards palliative care (PC) was favorable.

## Introduction

Palliative care is a multidisciplinary approach and is focused on both the patient and their family [1]. Commonly used terms such as supportive care, best supportive care, palliative care, and hospice care were rarely and inconsistently defined in the palliative oncology literature [2]. Sixty five percent of worldwide death was reported from non-communicable diseases (NCDs), i.e. cancer, diabetic mellitus, cardiovascular disease and chronic respiratory problem in which palliative care can play significant role in bringing relief for both physical and mental symptoms of the illness [3]. Palliative care includes the time range starting from the onset and progress of the chronic illness, through the terminal stages of the disease and until the end of life. It is a collaborative approach encompassing various managements, notably include medical and spiritual management [4]. Nurses are the main valuable palliative care team members who are responsible for the dimension of physical, functional, social, and spiritual patients' care [5]. The current expression of palliative care (PC) has developed to include patients who can live for several years with end-stage organ failure. PC is a care that promotes the quality of life of patients and their relatives fronting a problem linked with the life-threatening disease through avoidance and decreasing the suffering by means of early detection, perfect evaluation, and treatment of pain and other problems physical and nonphysical [6]. One activity authorized by this committee was a survey of nurses' knowledge of palliative care [7]. As death is an inevitable phenomenon that affects every human being; Nurses are present at both the beginning and the end of life, and play a key role; that role is seen as one of the most stressful condition of nursing [8, 9]. However, there is an obvious difference between nurses' qualification, experience, and training of palliative care towards Knowledge of PC [10].

Significant advances have been achieved in African palliative care providers to manage the highly prevalent and burdensome problems experienced by those with incurable terminal disease [11]. An essential factor affecting a successful implementation of PC is nurses' knowledge, and attitudes for providing care to dying patients [12, 13].Palliative care can be provided at any site including at patient's own home, health care facility, hospice unit, in hospital out-patient or daycare service [14]. There are several reports that show patients are dying in pain that could be treated, especially for patients with chronic illnesses. A consequence of pain being undertreated is that it could cost more money in health care than actually treating the pain [15].These avoidable experiences need to be improved so patients are able to live and die peacefully with in the absence of pain as much as possible [16]. A quantitative survey study was conducted in Saudi Arabia to identify nurses' attitude, knowledge and experiences on prioritizing palliative care in selected hospitals in Taif City. The result revealed that more than half of the nurses (62%) had poor knowledge regarding palliative care [13]. Due to the limited implementation of PC in Africa countries; too many patients do not get formal PC services [15]. Integrating palliative care education is required as the mainstay to improve students' knowledge and attitude [17]. There is still much to be done to improve the palliative care of patients with advanced incurable disease and particularly the care of patients during the terminal phase [18]. Therefore the aim of this study is to assess knowledge, attitudes and associated factors of nurses towards PC among nurses working in selected governmental hospitals in the Tigray region.

## Methods

Institutional based study design was conducted in Tigray governmental hospitals from September 2017 to June 2018. The Tigray region is located in the north part of Ethiopia and its capital city, Mekelle, is 782 km far from Addis Ababa. Tigray region covers an area of 54,569.25 square kilometers and its elevation is 600-2700 meters above sea level. The region has 2comprhensiveSpecialized hospitals, 15 General Hospitals, 1 military General Hospital, 204 Health Centers, 20 Primary Hospitals, and 712 satellite Health posts. Additional Curative and rehabilitative services are delivered by more than 500 private health facilities including hospitals, higher clinics, pharmacies, and rural drug vendors. There are three public universities and two public health science colleges in the region. There are 3864 nurses in the Region. The total population of Tigray region accounts 6,690,003.

All nurses who are working in Tigray governmental hospitals were Source population, all nurses working in randomly selected governmental hospitals in Tigray and who meet the inclusion criteria were also Study population. But nurses working in the central sterilization supply department, operating room, delivery rooms were excluded. The total sample size was allocated proportionally based on the number of nurses from each selected hospitals using simple random sampling. Then proportional allocation was done for each ward in each selected institution. Because of the sample size was less than 10,000, the sample size was determined using a Formula single population proportion. The sample size is calculated poor knowledge prevalence 30.5% in the previous study in Addis Ababa [19] with 5% marginal error, 95% and confidence interval (CI). Based on this assumption, with the none-response, rate 10% total sample size was 355.

A self-administered English questionnaire was used for data collection. The knowledge questions adopted from the Palliative Care Quiz for Nursing (PCQN). The attitude scale adopted from Frommelt's Attitude toward Care of the Dying (FATCOD) and modified so as to make it fit the Ethiopia context [19]. The tool not translated to local language because the study participants are health professionals. The data collection instrument contains three sections. Six-degree nurses as data collectors from those randomly not selected hospitals and two master nurses as supervisor was selected who have an experience of data collection. Data quality control was controlled by pretest in 5% of the sample nurses in St. Mery hospital. Two full-day training was given for data collectors and supervisor regarding the study, the questionnaire and data collection procedure by the principal investigator. The data was checked by supervisors and principal investigators for its clarity and completeness. Data was kept in the form of the file in the secure place where no one can access it except the investigator.

Data was entered into Epi-info and export to SPSS Version 22 and check for missing values. After data entry cleaning was computed by running frequency. Descriptive statics was used to describe frequency and percentages and displayed in tables and text. Binary logistic regression was done to see the crude significant relation of each independent variable with dependent variables. Then independent variables found significant entered to multivariate logistic regressions to control the effect of confounding. Finally, significant factors were identify based on AOR include in 95% confidence level at P-value less than 0.05.

**Dependent variables:** knowledge of palliative care, attitude on palliative care

**Independent variables:** work institution, Age, level education, ward, experience, Experience in the care of the chronically ill patient, palliative care training and duration of training. This study operational zed the variables as follows: **Good knowledge** = ≥ 75% of the total score of the Palliative Care Quiz for Nursing (PCQN) scale. **Poor knowledge** = < 75% of the total score of the PCQN scale [20]. **Favorable attitude** = ≥ 50% of the total score of Frommelt Attitude toward Care of the Dying (FATCOD) Scale. **Unfavorable attitude** = < 50% of total score of the FATCOD Scale [20].

## Results

**Socio-demographic characteristics of nurses:** the total number of participants was 355 with the response rate was 100%. The number of participants by hospital were from lemlem Karl hospital 49 (13.8%), Mekelle hospital 95 (26.8%),Wukro hospital 38 (10.7%), Abi Adi hospital 49 (13.8%),Aksum comprehensive specialized hospital 67(18.9%) and kahsay abera hospital 57 (16.1%). The majority of the participants 206 (58%) were female and the mean age of the respondents was 30.66 years ± 7.80 SD (range from 21 to 55). Regarding training, the majority of nurses 267(75.2%) were not trained about palliative care. out of eighty eight (24.8%) trained nurses; 63 (17.7%) 1-2 weeks and 25(7.0%) 6 months taken (**Table 1**).

**Table 1:** Socio-demographic characteristics of nurses at selected hospitals in Tigray region,2018

Variables	Frequency No (355)	Percentage% (100)
**work institution lemlem karl hospital**	49	13.8
Mekelle hospital	95	26.8
Wukro hospital	38	10.7
AbiAdi hospital	49	13.8
AKCSH	67	18.9
Kahsay abera hospital	57	16.1
**age of nurses20-30**	195	54.9
31-40	102	28.7
41-50	50	14.1
50+	8	2.3
**Educational level diploma**	169	47.6
degree	186	52.4
**ward/work area medical ward**	74	20.8
surgical ward	63	17.7
recovery ward	41	11.5
ICU	44	12.4
OPD	33	2.3
pediatric ward	31	8.7
emergency	33	9.3
neonatal ward	28	7.9
other	8	9.3
**work experience less than 5 years**	136	38.3
5-10 years	105	29.6
10-15	94	26.5
greater than 15 years	20	5.6
**Experience in caring terminally ill patient**		
Daily	165	46.5
Once per week	79	22.3
Never	54	15.2
Few times per year	40	11.3
Once per month	17	4.8
**Training**		
yes	267	75.2
no	88	24.8
**How long**		
1-2 weeks	63	17.7
6 month	25	7.0
Never	267	75.2

**Nurses' knowledge towards PC:** nearly 89.9% of the respondents knew the definition of PC and 80.6% agreed that PC is being given when patient's conditions are downhill trajectory or deterioration. Similarly, 86.5% of nurses responded that the extent of the disease determines the method of pain treatment. In addition, Drug addiction was a major problem when morphine is used on a long-term basis for the management of pain 289(81.4%).Forty-nine percent of the subjects agreed that accumulation of losses renders burn out for those who work in PC. Of the total respondents 77.5%, 72.4%, 72.7% agreed that adjuvant therapies are important in pain management, that the patients right not to resuscitate (DNR) should be respected, and that terminally ill patients should be supported to have hope, orderly. Only two hundred twenty-three (62.8%) had good knowledge out of the whole study participants, towards PC (Table 2).

**Table 2:** Distributions of nurses’ knowledge towards palliative care at selected hospitals in Tigray region, March, 2018

No	Variables	Yes N (%)	No N (%)	Don’t Know N (%)
1	Do you know the definition palliative care?	319 (89.9)	7 (2.0)	29(8.2)
2	Palliative care is only appropriate in situations of a downhill trajectory or deterioration in conditions.	286 (80.6)	47 (13.2)	22(6.2)
3	The extent of the disease determines the method of pain treatment.	307 (86.5)	35 (9.9)	13(3.7)
4	Adjuvant therapies are important in managing pain.	275 (77.5)	22 (6.2)	58(16.3)
5	Drug addiction is a major problem when morphine is used on a long-term basis for the management of pain.	289 (81.4)	39 (11.0)	27(7.6)
6	The provisions of palliative care require emotional detachment	126 (35.5)	213 (60.0)	16(4.50
7	During the terminal stages of an illness, drugs that can cause respiratory depression are appropriate for the treatment of severe dyspnea.	98 (27.6)	133 (37.5)	124(34.9)
8	The philosophy of palliative care is compatible with that of aggressive treatment.	122 (34.4)	169 (47.6)	64(18.0)
9	The use of placebos is appropriate in the treatment of some types of pain.	190 (53.5)	102(28.7)	63(17.7)
10	Meperidine (Demerol^®^) is not an effective analgesic for the control of chronic pain.	89 (25.1)	152(42.8)	114(32.1)
11	The accumulation of losses renders burnout Inevitable for those who work in palliative care.	174 (49.0)	70(19.7)	111(31.3)
12	Manifestations of chronic pain are different from those of acute pain.	280 (78.9)	59(16.6)	16(4.5)
13	Terminally ill patients have the right to choose “Do not resuscitate” (DNR).	257 (72.4)	60(16.9)	38(10.7)
14	Terminally ill patients should be encouraged to have hope against all odds.	258 (72.7)	77(21.7)	20(5.6)

**Distribution of nurse's attitude according to the degree of agreement towards items of FATCOD:** more than half of the participant nurses 217(61.1%) strongly disagree that as a patient nears death; the nurse may withdraw from his/her participation. In contrast majority of the respondents, 197(55.5%) Giving nursing care to the chronically sick patient is a worthwhile learning experience was agreed. One hundred eighty-nine agreed Families should be concerned about helping their dying member make the best of his/her remaining life. On the other hand, over half of the nurses 210(59.2%) and 214(60.3%) Strongly disagreed that nursing care should extend to the family of the dying person, would be uncomfortable talking about impending death with the dying Person respectively. In general, more than half of the respondent 200 (56.3%) had a favorable attitude towards PC (Table 3).

**Table 3:** Distribution of nurse’s attitude according to their degree of agreement toward items of FATCOD at selected hospitals in Tigray region, 2018

No	Statement	SD (%)	D (%)	U (%)	A (%)	SA (%)
1	Palliative care is given only for dying patient.	139(39.2)	143(40.3)	29(8.2)	21(5.9)	23(6.5)
2	As a patient nears death; the nurse should withdraw from his/her involvement.	217(61.1)	86(24.2)	21(5.9)	13(3.7)	18(5.1)
3	Giving nursing care to the chronically sick patient is a worthwhile learning experience.	45(12.7)	13(3.7)	23(6.5)	197(55.5)	77(21.7)
4	It is beneficial for the chronically sick person to verbalize his/her feelings.	26(7.3)	24(6.8)	28(7.9)	176(49.6)	101(28.5)
5	Family members who stay close to a dying person often interfere with a professionals' job with the patient.	99(27.9)	61(17.2)	30(8.5)	112(31.5)	53(14.9)
6	The length of time required to give nursing care to a dying person would frustrate me.	84(23.7)	124(34.9)	24(6.8)	88(24.8)	35(9.9)
7	Families should be concerned about helping their dying member make the best of his/her remaining life.	16(4.5)	13(3.7)	20(5.6)	189(53.2)	117(33.0)
8	Family should maintain as normal an environment as possible for their dying member.	20(5.6)	23(6.5)	38(10.7)	184(51.8)	90(25.4)
9	The nurse should not be the one to talk about death with the dying person.	77(21.7)	77(21.7)	33(9.3)	120(33.8)	48(13.5)
10	The family should be involved in the physical care of the dying person.	53(14.9)	62(17.5)	22(6.2)	128(36.1)	90(25.4)
11	It is difficult to form a close relationship with the family of a dying member.	63(17.7)	107(30.1)	34(9.6)	82(23.1)	69(19.4)
12	There are times when death is welcomed by the dying person.	31(8.7)	71(20.0)	30(8.5)	129(36.3)	94(26.5)
13	Nursing care for the patient's family should continue throughout the period of grief and bereavement.	60(16.9)	108(30.4)	35(9.9)	91(25.6)	61(17.2)
14	The dying person and his/her family should be the in-charge decision makers.	174(49.0)	119(33.5)	25(7.0)	19(5.4)	18(5.1)
15	Addiction to pain relieving medication should not be a nursing concern when dealing with a dying person.	65(18.3)	105(29.6)	26(7.3)	78(22.0)	81(22.8)
16	Nursing care should extend to the family of the dying person.	210(59.2)	72(20.3)	20 (5.6)	36(10.1)	17(14.8)
17	When a patient asks, ‘Nurse am I dying?’ I think it is best to change the Subject to something cheerful.	70(19.7)	105(29.6)	32(9.0)	78(22.0)	70(19.7)
18	I am afraid to become friends with chronically sick and dying patients.	110(31.0)	109(30.7)	42(11.8)	54(15.2)	40(11.3)
19	I would be uncomfortable if I entered the room of a terminally ill person and found him/her crying.	62(17.5)	106(29.9)	36(10.1)	86(24.2)	65(18.3)
20	I would be uncomfortable talking about impending death with the dying Person.	214(60.3)	88(24.8)	23(6.5)	14(3.90	16(4.5)
21	It is possible for nurses to help patients prepare for death.	100(28.2)	94(26.5)	42(11.8)	61(17.2)	58(16.3)
22	Death is not the worst thing that can happen to a person.	149(42.0)	117(33.0)	25(7.0)	37(10.4)	27(7.6)
23	I would feel like running away when the person actually died.	137(38.6)	121(34.1)	21(5.9)	42(11.8)	34(9.6)
24	I would feel like running away when the person actually died.	129(36.3)	119(33.5)	23(6.5)	56(15.8)	28(7.9)

**Association between socio-demographic data and nurses' knowledge towards palliative care:** ward, training, and duration of training had a significant association with knowledge of nurses on palliative care; however, institution, age, gender, level of education, work experience and experience of caring for terminally ill patient did not. Respondents with the medical ward had more knowledgeable (AOR = 3.413, CI = 1.388-8.392, P = 0.019) than recovery ward. Nurses who had training on PC had approximately greater knowledge (AOR=3.488; CI=1.735-7.015; P=0.00) than those who had never training (Table 4).

**Table 4:** The association of socio-demographic characteristics and knowledge of nurses towards PC at selected hospitals in Tigray, June 2018

variables		knowledge		P value (x2)	COR 95(CI)	AOR 95(CI)
		Good n (%)	Poor n (%)			
work institution	Lemlem karl hospital	32(65.3)	17(34.7)	0.180		
	Mekelle hospital	55(57.9)	40(42.1)			
	Wukro hospital	30(78.9)	8(21.1)			
	Abi adi hospital	26(53.1)	23(46.9)			
	AKCSH	43(64.2)	24(35.8)			
	kahsay abera hospital	37(64.9)	20(35.1)			
age of nurses	20-30	111(61.3)	70(38.7)	0.248		
	31-40	64(61.0)	41(39.0)			
	41-50	38(65.5)	20(34.5)			
	50+	10(90.9)	1(9.1)			
Educational level	diploma	100(59.2)	69(40.8)	0.176		
	degree	123(66.1)	63(33.9)			
ward/work area	medical ward	50(67.6)	24(32.4)	0.011	1	1
	surgical ward	39(61.9)	24(75.0)		1.282(.634, 2.592)	1.187(.577,2.439)
	pediatric ward	23(56.1)	18(43.9)		1.630(.743, 3.577)	1.537(.688,3.435)
	ICU	31(70.5)	13(29.5)		.874(.389,1.964)	.815(.356,1.866)
	OPD	26(78.8)	7(21.2)		.561(.213,1.474)	.466(.175,1.240)
	recovery ward	12(38.7)	19(61.3)		3.299(1.380,7.884)	3.413(1.388,8.392)
	emergency	20(60.6)	13(39.4)		1.354(.578,3.172)	1.438(.598,3.454)
	neonatal ward	20(71.4)	8(28.6)		.833(.321,2.162)	.814(.307,2.153)
	other	2(25.0)	6(38.1)		6.25(1.173,33.290)	8.24(1.425,47.733)
work experience	less than five years	86(63.2)	50(36.8)	0.093		
	5-10 years	57(54.3)	48(45.7)			
	10-15 years	64(68.1)	30(31.9)			
	greater than 15 years	16(80.0)	4(20. 0)			
Experience in caring terminally ill patient	Daily	107(64.8)	58(35.2)	0.524		
	Once per week	45(57.0)	34(43.0)			
	Once per month	32(59.3)	22(40.7)			
	Few times per year	29(72.5)	11(27.5)			
	Never	10(41.2)	7(58.8)			
Training	Yes	69(78.4)	19(21.6)	0.000	1	1
	no	154(57.7)	113(42.3)		2.665(1.518,4.678)	3.488(1.735,7.015)
How long	1-2 weeks	50(79.4)	13(20.6)	0.000	1	1
	6 month	19(76.0)	6(24.0)		1.215(.403, 3.657)	1.476(.471,4.621)

**Association between socio-demographic variables and nurses attitude towards PC:** Work institution, the age of nurses, training, and duration of training had a significant association with the attitude of nurses. There were no statistically significant relationships between Educational level, ward/work area, work experience and experience in caring terminally ill patient. In addition, our findings revealed that nurses working in the lemlem Karl hospital had more than twice favorable attitude towards palliative care (AOR = 2.541; CI 1.106-5.835; p = 0.013) compared to kahsay Abera Hospital. Similarly, nurses who had a 20-30 years ago had revealed unfavorable attitude [AOR = 2.660; CI 1.386-5.106; p = 0.002] compared to those who held 50+ years. Concerning training nurses trained on PC had a more favorable attitude towards PC compared to the nurse who did not take PC training (AOR = 3.472; CI 1.750-6.888; P = 0.00) (Table 5).

**Table 5:** The association of socio-demographic characteristics and attitude of nurses towards palliative care at selected hospitals in Tigray, March, 2018

variables		Attitude		P-value (x^2^)	COR 95%(CI)	AOR 95%(CI)
		Favorable n (%)	Unfavorable n (%)			
work institution	lemlem karl hospital	27(55.1)	22(44.9)	0.006(0.010)	0.407(.185,.895)	1
	Mekelle hospital	62(65.3)	33(34.7)		0.266(.133,.533)	0.601(.286,1.264)
	Wukro hospital	23(60.5)	15(39.5)		0.326(.139,.765)	0.691(.273,1.746)
	Abi Adi hospital	29(59.2)	20(40.8)		0.345(.156,.762)	0.892(.380,2.092)
	AKCSH	40(59.7)	27(66.7)		0.338(.162,.705)	0.765(.349,1.677)
	kahsay abera hospital	19(33.3)	38(40.3)		1	2.541(1.106,5.835)
age of nurses	20-30	112(61.9)	69(38.1)	0.013 (0.002)	0.137(.029,.652)	1
	31-40	60(57.1)	45(42.9)		0.167(.034,.809)	1.456(.860,2.464)
	41-50	26(44.8)	32(55.2)		0.274(.054,1.378)	2.660(1.386,5.106)
	50+	2(18.2)	9(81.8)		1	13.6(2.576,72.574)
Educational level	diploma	89(52.7)	80(47.3)	0.183		
	degree	111(59.7)	75(40.3)			
ward/work area	medical ward	44(59.5)	30(40.50	0.285		
	surgical ward	36(57.1)	27(42.9)			
	pediatric ward	18(43.9)	23(56.1)			
	ICU	32(72.7)	12(27.30)			
	OPD	18(54.5)	15(51.5)			
	recovery ward	18(58.1)	13(41.9)			
	emergency	16(48.5)	17(45.5)			
	neonatal ward	13(46.4)	15(53.6)			
	other	5(62.5)	3(37.5)			
work experience	<5 years	80(58.8)	56(41.2)	0.111		
	5-10 years	60(57.1)	45(42.9)			
	10-15 years	54(57.4)	40(42.6)			
	> 15 years	6(30.0)	14(70.0)			
Experience in caring terminally ill patient	Daily	89(53.9)	76(46.1)	0.273		
	Once per week	45(57.0)	34(43.0)			
	Once per month	28(51.9)	26(48.1)			
	Few times per year	29(72.5)	11(27.5)			
	Never	9(52.9)	8(47.1)			
Training	Yes	61(69.3)	27(30.7)	0.005	0.481(.288,.803)	1
	no	139(52.1)	128(47.9)			3.472(1.750,6.888)
How long	1-2 weeks	48(76.2)	15(23.8)	0.01(.003)	0.339(.181,.636)	4.611(1.589,13.384)
	6 month	13(52.0)	12(48.0)		1.002(.441,2.277)	
	Never	139(52.1)	128(47.9)		1	

* Significant P≤ 0.05 level

## Discussion

Nearly 89.9% of the respondents knew the definition of PC and 80.6% agreed that PC is being given when patient's conditions are downhill trajectory or deterioration. Similarly, 86.5% of nurses responded that the extent of the disease determines the method of pain treatment. This is similar to the study done in southeast Iran even if the percentage is slightly higher in the present study [21] and Addis Ababa[19]. The possible reason might be due to the similarity of study design in this study. The result of this study showed that the majority of nurses had good knowledge 62.8% towards PC. But it is less than the study conducted in India (79.5%) [22]. In contrast it is higher than the studies done in DR Congo (29.5%) [23], Greece (26%) [24] and Addis Ababa (30.5%) [19]. The possible reason for this might be due to the fact that PC educational level was improved in each institution.

The findings from this study had also confirmed the strong association between the type of wards, training on attitude towards PC and duration of training. Though some studies showed that age, past and present experience with death, education regarding the end of life care and year of clinical experience had a significant influence on one's knowledge towards PC [24]. Regarding attitude in this study the majority, 56.3%, of nurses had a favorable attitude towards PC, which is also evident in other studies Egypt (56.6%) [3], Zimbabwe 56% [25], Addis Ababa 76% [19] and DR Congo (58.9% [23]. This study is not in line with the study done in Venjaramoodu(79.5%) [20], Taif City, in Saudi Arabia, For this, 83% of the study respondents have a positive attitude regarding palliative care [13] whereas the study done in Udupi district , Indian showed that 92.8% of nurses had favorable attitude (56.7± 8.5) towards palliative care [22].

The possible reason for this difference may be due to the presence of curriculum education content about palliative care in Udupi district or absence (inadequacy) palliative care education in Ethiopia. But the present study attitude of nurses less than the previous study in Addis Ababa which was 76%, of nurses had a favorable attitude towards palliative care [26].This difference may be due to the Participants' educational preparation because the first-degree level educated nurses were 85.5% in the current study only 52.4%. So that holding first-degree nursing might be able to understand the FATCOD scale in a better way than that of diploma holder. Moreover, this study is higher than in another study in Addis Ababa to assess the attitude of nurses' and barriers regarding cancer pain management at selected health institutions offering cancer treatment which showed 53.7%, of the nurses', have a negative attitude, towards cancer pain management [27]. The possible difference may be due to the present study is wider in scope than the previous one as well as due to the instrument variation.

## Conclusion

The result of this study suggested that the majority of respondents that have had a poor knowledge towards PC but attitude were favorable. Similarly, work institution, the age of nurses and duration of training on PC were significantly associated with knowledge; institution, duration of training in additional training on pc, on the other hand, were found to be significant finding with the PC attitude. In conclusion, much should be done to assist nurses to perform their duties based on the knowledge they grasp in various training, workshops, formal or informal education. The curriculum designer and policymaker in Ethiopia should also integrate courses related to PC issues so as to improve their graduates' level of knowledge.

### What is known about this topic

- Inadequate palliative care service implementation was delivered in Ethiopia;
- Although large number of patients suffer from pain in the last stage of life there is no standard palliative care service.

### What this study adds

- Nurses have poor knowledge towards PC but attitude were favorable;
- Work institution, the age of nurses and duration of training on PC were significantly associated with knowledge; institution, duration of training in additional training on palliative care were found to be significant.

## Competing interests

The authors declare no competing interests.

## References

[1] Davison SN (2010). End-of-life care preferences and needs: perceptions of patients with chronic kidney disease. Clin J Am Soc Nephrol.

[2] Thomas N, Nixen N, Ramshida T, Gireesh G (2015). A descriptive study to assess the knowledge and attitude of staff nurses regarding palliative care in selected hospital, mangaluru. American International Journal of Research in Humanities, Arts and Social Sciences.

[3] Hui D, De La Cruz M, Mori M, Parsons HA, Kwon JH, Torres-Vigil I (2013). Concepts and definitions for “supportive care”, “best supportive care”, “palliative care” and “hospice care” in the published literature, dictionaries, and textbooks. Supportive Care in Cancer.

[4] Julie NK, Simon IK, Charles MM, Irène KU, Mahuridi A, Narcisse MK (2017). Factors Associated with the Implementation of the Nursing Process in the Public Hospitals of Lubumbashi in the Democratic Republic of Congo: A Cross-Sectional Descriptive Study. Open Access Library Journal.

[5] Foster TL, Lafond DA, Reggio C, Hinds PS (2010). Pediatric palliative care in childhood cancer nursing: from diagnosis to cure or end of life. Seminars in oncology nursing.

[6] Repolarization ECIM (2013). Knowledge, attitude and practice of medical ethics of faculty of a medical university in Karachi, Pakistan. ESPNIC 2013.

[7] Proctor M, Grealish L, Coates M, Sears P (2000). Nurses' knowledge of palliative care in the Australian Capital Territory. Int J Palliat Nurs.

[8] Harding R, Selman L, Agupio G, Dinat N, Downing J, Gwyther L (2010). Validation of a core outcome measure for palliative care in Africa: the APCA African Palliative Outcome Scale. Health and Quality of Life Outcomes.

[9] Mwangi-Powell F, Dix O (2011). Palliative care in Africa: an overview. Africa Health.

[10] Bing-Jonsson PC, Bjørk IT, Hofoss D, Kirkevold M, Foss C (2015). Competence in advanced older people nursing: development of ‘Nursing older people-Competence evaluation tool’. International journal of older people nursing.

[11] Organization WH (2014). Strengthening of palliative care as a component of integrated treatment throughout the life course. Journal of Pain & Palliative Care Pharmacotherapy.

[12] Sorifa B, Mosphea K (2015). Knowledge and practice of staff nurses on palliative care. IJHRMLP.

[13] Youssef H, Mansour M, Al-Zahrani S, Ayasreh I, Abd El-Karim R (2015). Prioritizing palliative care: assess undergraduate nursing curriculum, knowledge and attitude among nurses caring end-of-life patients. European Journal of Academic Essays.

[14] Burns M, McIlfatrick S (2015). Palliative care in dementia: literature review of nurses' knowledge and attitudes towards pain assessment. International journal of palliative nursing.

[15] Ferrell BR, Grant M, Virani R (1999). Strengthening nursing education to improve end-of-life care. Nursing Outlook.

[16] Bittel N, Neuenschwander H, Stiefel F (2002). “Euthanasia”: a survey by the Swiss Association for Palliative Care. Supportive care in cancer.

[17] Matzo M, Emanual E (1997). Oncology nurses' practices of assisted suicide and patient-requested euthanasia. Oncology nursing forum.

[18] Ventafridda V (2006). According to the 2002 WHO definition of palliative care.

[19] Zewdu F, Kassa H, Hailu M, Murugan R, Woldeyohannes D (2017). Knowledge, attitude and practice and associated factors towards palliative care among nurses working in selected hospitals, Addis Ababa, Ethiopia. European Journal of Cancer.

[20] Manwere A, Chipfuwa T, Mukwamba MM, Chironda G (2015). Knowledge and attitudes of registered nurses towards pain management of adult medical patients case of Bindura hospital.

[21] Iranmanesh S, Razban F, Tirgari B, Zahra G (2014). Nurses' knowledge about palliative care in Southeast Iran. Palliative & supportive care.

[22] Karkada S, Nayak BS, Malathi (2011). Awareness of palliative care among diploma nursing students. Indian journal of palliative care.

[23] Mukemo AK, Kasongo NM, Nzaji MK, Tshamba HM, Mukengeshayi AN, Nikulu JI Assessment of Nurses' Knowledge, Attitude and Associated Factors towards Palliative Care in Lubumbashi's Hospitals.

[24] Maria K, Evanthia V, Petros KA, Dimitris N (2016). Assessment of Knowledge and Associated Factors towards Palliative Care among Greek Nurses. Assessment.

[25] Grant L, Downing J, Namukwaya E, Leng M, Murray SA (2011). Palliative care in Africa since 2005: good progress, but much further to go. BMJ Supportive & Palliative Care.

[26] Das AG, Haseena T (2015). Knowledge and attitude of staff nurses regarding palliative care. Int J Sci Res.

[27] Kassa RN, Kassa GM (2014). Nurses' Attitude, Practice and Barrier s toward Cancer Pain Management, Addis Ababa, Ethiopia. J Cancer Sci Ther.

